# Establishment of a Novel Human Endometrial Organoid

**DOI:** 10.1002/rmb2.70030

**Published:** 2026-02-16

**Authors:** Toshihide Yoneda, Isao Tamura, Azumi Yoshimura, Marina Ito, Amon Shiroshita, Taishi Fujimura, Yuichiro Shirafuta, Shun Sato, Norihiro Sugino

**Affiliations:** ^1^ Department of Obstetrics and Gynecology Yamaguchi University Graduate School of Medicine Ube Japan; ^2^ Yamaguchi University Graduate School of Medicine Ube Japan

**Keywords:** apical‐out, ECM‐free, endometrial organoid, endometrium, implantation

## Abstract

**Purpose:**

Human implantation is difficult to study in vivo because of ethical constraints, making the development of in vitro models essential for mechanistic investigations. Here, we applied the technique previously established in mice to develop a novel human endometrial organoid.

**Methods:**

Human endometrial tissues were digested and cultured as adherent monolayers without extracellular matrix (ECM). After 10 days, self‐organized aggregates appeared and were transferred to low‐attachment plates for three‐dimensional culture. To assess hormonal responsiveness, organoids were treated with medroxyprogesterone acetate and cyclic adenosine monophosphate for 4 days.

**Results:**

The resulting organoids consisted of EpCAM‐positive epithelial cells forming an outer layer and PDGFRα‐positive stromal cells occupying the inner region. MUC1 and acetylated α‐tubulin, proteins localized on the apical surface of endometrial epithelial cells in vivo, were expressed on the outer surface of the epithelial layer in our organoids. Hormonal stimulation altered the expression of receptivity markers, including increased PAEP and decreased PGR in epithelial cells and increased FOXO1 in both epithelial and stromal cells, reflecting in vivo implantation‐phase responses.

**Conclusion:**

We established a novel ECM‐free human endometrial organoid that recapitulates key structural and hormonal characteristics of the human endometrium, providing a promising platform for studying human implantation mechanisms.

## 1 Introduction

Implantation is a complex, stepwise event in which a blastocyst first adheres to the luminal epithelium of the endometrium and subsequently invades the underlying layer of decidualized stromal cells, ultimately leading to placental formation [1]. This process depends on a finely orchestrated crosstalk between the embryo and the maternal endometrium [1]. Although assisted reproductive technologies (ART), including in vitro fertilization and intracytoplasmic sperm injection, have advanced remarkably in recent years, recurrent implantation failure remains a major unresolved cause of infertility, affecting approximately 10%–15% of ART patients [2]. Effective therapeutic approaches are still lacking, largely because the mechanisms underlying human implantation remain poorly understood [1], this is mostly due to the ethical difficulties of analyzing the implantation process in vivo. Therefore, studies on implantation have relied primarily on rodent models [3]. However, significant interspecies differences, such as the orientation of embryo attachment, the depth of trophoblast invasion, and the molecular regulation of stromal decidualization [4], limit the extrapolation of mouse data to the human context. Consequently, there is a strong demand for a physiologically relevant in vitro system that can faithfully reproduce the human implantation process.

Early attempts to develop in vitro implantation models placed blastocysts or trophoblast cell aggregates onto two‐dimensional (2D) monolayers of endometrial epithelial cells (EECs) or stromal cells (ESCs) [5, 6]. Later, layered three‐dimensional (3D) co‐cultures of EECs and ESCs were developed to mimic the 3D structure of the endometrium [7]. Nevertheless, these approaches failed to reconstruct the spatial and functional complexity of the endometrial structure [8]. With the advent of organoid technology, several human tissues have been successfully modeled in vitro [8, 9, 10]. Human endometrial organoids have been established as a novel in vitro 3D culture model of EECs [11] and were expected to be promising for implantation studies. However, epithelial‐only organoids have several limitations. First, they exhibit a basal‐out/apical‐in polarity [11], in which the apical surface faces the organoid lumen. Thus, simple co‐culture with blastocysts cannot establish direct contact. To enable attachment, the blastocyst must be microinjected into the lumen, which is artificial and nonphysiological. Second, the organoids lack ESCs beneath the epithelium, so they cannot reproduce the invasion of blastocysts into the stromal compartment. Moreover, not only EECs but also ESCs undergo marked morphological and functional differentiation during implantation, a process known as decidualization, which is crucial for successful implantation [12, 13, 14, 15, 16, 17]. Therefore, to faithfully reproduce implantation in vitro, an organoid that incorporates both epithelial and stromal components is essential. Furthermore, EECs and ESCs must also retain hormone responsiveness, allowing them to undergo implantation‐phase–specific changes, including decidualization. Finally, conventional organoids are cultured within an extracellular matrix (ECM) such as Matrigel [11, 18], which also prevents the blastocyst from directly contacting the EECs, even if they are co‐cultured.

Although several human endometrial‐like 3D structures have been reported [18, 19, 20], none have simultaneously achieved apical‐out epithelial polarity, inclusion of stromal cells, hormone responsiveness, and ECM‐free. Recently, we established a novel mouse endometrial organoid culture system that fulfilled these criteria and successfully reproduced implantation in vitro through co‐culture with mouse blastocysts [21]. In this study, extending the methodology established in mice, we developed a hormone‐responsive, ECM‐free human endometrial organoid with apical‐out epithelial polarity and an inner stromal compartment, providing a new platform for studying human implantation in vitro.

## 2 Methods

### 2.1 Patient Samples

Human endometrial tissue samples were obtained from patients at Yamaguchi University Hospital. The study protocol was reviewed and approved by the Institutional Review Board of Yamaguchi University Hospital (approval no. H26‐102‐9) and was conducted in accordance with the latest revision of the Declaration of Helsinki. Written informed consent was obtained from all participants before sample collection. All patients had not received any hormonal treatment before sample collection.

### 2.2 Organoid Culture From Human Endometrium

The protocol to generate the endometrial organoid was primarily based on the previously reported method [21], with minor modifications as described below. Human endometrial tissues at various menstrual phases were collected from patients who underwent hysterectomy at Yamaguchi University Hospital. Detailed information on the patients' backgrounds is provided in Table S1. Endometrial tissue was obtained using a Cooper and curette after hysterectomy or collected from exfoliated endometrial tissue during endometrial polypectomy. The collected tissues were immediately placed in Dulbecco's Modified Eagle Medium (Thermo Fisher Scientific: Cat#31053–028) and transported on ice to the laboratory. The collected endometrial tissues were extensively rinsed with cold PBS (FUJIFILM: Cat#166–23555). Large blood clots were carefully removed under a stereomicroscope, and the tissues were minced into approximately 2 mm fragments. The tissues were incubated with 1 mg/mL collagenase (Sigma‐Aldrich: Cat#C5138) and 0.1 mg/mL DNase I (Sigma‐Aldrich: Cat#10104159001) in Ca2+/Mg2 + −free PBS (FUJIFILM: Cat#166–23555) for 30 min at 37°C water bath with gentle shaking at 100 bpm. After adding cold PBS to a final volume of 15 mL, the isolation of epithelial fractions was performed as previously described [21, 22, 23]. Tissue fragments were mechanically dissociated by pipetting up and down at least 10 times and then allowed to settle down under normal gravity for 1 min. Since the supernatant contains the epithelial fraction [23], it was collected. This process was repeated twice. After centrifuging the supernatant at 200 g for 3 min at 4°C, the pellet was resuspended in basal medium {advanced DMEM (Thermo Fisher Scientific: Cat#12634010) with Glutamax (0.2 mM; Thermo Fisher Scientific: Cat#35050061), HEPES (1 mM; Thermo Fisher Scientific: Cat#15630080)}and centrifuged twice. Finally, the pellet was resuspended with an expansion medium [22] {advanced DMEM supplemented with penicillin/streptomycin (1%; FUJIFILM: Cat#161–23181), Glutamax (2 mM), B27 (2%; Thermo Fisher Scientific: Cat#17504044), N2 (1%; Thermo Fisher Scientific: Cat#17502048), insulin‐transferrin‐selenium (ITS, 1%; Thermo Fisher Scientific: Cat#41400–045), HEPES (10 mM), nicotinamide (1 mM; Thermo Fisher Scientific: Cat#N0636), EGF (50 ng mL^−1^; Thermo Fisher Scientific: Cat#PMG8043), FGF‐Basic (50 ng mL^−1^; FUJIFILM: Cat#06205181), Noggin (100 ng mL^−1^; Thermo Fisher Scientific: Cat#120‐10C), the TGFβ/Alk inhibitor A83‐01 (0.5 μM; Thermo Fisher Scientific: Cat#2939/10), R‐spondin (200 ng mL^−1^; Thermo Fisher Scientific: Cat#51996611), WNT3A (200 ng mL^−1^; Proteintech Group Inc.: Cat# HZ‐1296), 17β‐Estradiol(E_2_) (10^−8^ M; SIGMA: Cat#E2758)} with Y27632 (10 μm; MedChemExpress: Cat#HY‐10583) up to 3 mL and kept on ice as the epithelial fraction until the further processing with stromal fraction. The remaining pellet, from which the supernatant with the epithelial fraction had been removed, was further digested with 1 mg/mL collagenase and 0.1 mg/mL DNase I for 50 min, and filtered through a 70 μm cell strainer (Falcon: Cat#352350) followed by a centrifuge at 500 g for 5 min at 4°C. The pellet, designated as stromal fraction, was resuspended in basal medium and washed twice. The number of epithelial glands in the epithelial fraction was counted under a microscope. To standardize the input cell composition for organoid formation, 3000, 6000, or 10 000 epithelial glands were combined with their corresponding stromal fractions in a volume proportional to the number of epithelial glands used (e.g., when half of the epithelial glands in the epithelial fraction were used, half of the stromal fraction collected in parallel was added), ensuring a consistent epithelial–stromal ratio for organoid generation. Organoids were generated using more than 6000 epithelial glands and the corresponding stromal fraction unless otherwise specified. The mixed tissue was seeded onto a 24‐well culture plate (Falcon: Cat#353047) with 1 mL of expansion medium without any coating. Cells were incubated at 37°C in a 5% CO₂ atmosphere, and the expansion medium was changed on days 1, 3, 5, 7, and 9. On day 1, the dishes were gently washed twice with basal medium to remove non‐adherent cells and then replaced with expansion medium. After washing, epithelial and stromal cells began to aggregate naturally in situ. In samples derived from the early proliferative phase, epithelial and stromal cells dynamically aggregated after washing, forming a single large aggregate. In such cases, following the organoid generation protocol we previously established in mice [21], the large aggregate was collected with a 1000‐μL pipette tip into a 1.5‐mL tube, and gently dissociated by pipetting with a slightly trimmed 200‐μL tip. The small aggregates were reseeded into the same culture dish, which become small aggregates. On day 10, each aggregate was picked up and individually transferred into a 96‐well low‐attachment U‐bottom dish (Sumitomo Bakelite: Cat#MS‐9096 U) containing expansion medium. The medium was changed every other day. After 3 days of suspension culture (day 13), the organoids were used for subsequent experiments. We attempted organoid generation from endometrial tissues obtained from 20 patients, and organoids were successfully established in 18 cases, yielding an establishment rate of 90%.

### 2.3 Hormonal Treatment

Organoids were treated for 4 days with medroxyprogesterone acetate (MPA, 10^−6^ M) and 3′,5′‐cyclic adenosine monophosphate (cAMP, 0.5 mM). These concentrations and treatment period were determined according to commonly used conditions for inducing the implantation phase and decidualization in vitro [12, 16]. The control group was cultured under the same conditions without cAMP or MPA. The expansion medium, with or without hormones, was gently replaced every other day.

### 2.4 Immunofluorescence Staining

Immunofluorescence staining of cells under adherent cultures was performed after tissue and cell clearing using CUBIC reagent with the manufacturer's protocol [21, 24, 25]. Briefly, the medium was removed from each tissue, followed by the addition of 4% paraformaldehyde and fixation for 24 h at 4°C. Tissues were pretreated with CUBIC‐L (TOKYO CHEMICAL INDUSTRY: Cat#T3740) for 2 days at 37°C and then thoroughly washed with PBS. Subsequently, nuclei were stained with DAPI using the CUBIC‐HVTM1 3D nuclear staining kit (TOKYO CHEMICAL INDUSTRY: Cat#C3709). Tissues were incubated with primary antibodies (Table S2) for 2 days at room temperature with gentle shaking. Then, they were washed with PBS three times for 2 h each and incubated with secondary antibodies (Table S2) for 1 day at room temperature with gentle shaking. Post‐fixation was performed with 1% formaldehyde, followed by washing with PBS. Tissues were incubated in 50% CUBIC‐R+ (TOKYO CHEMICAL INDUSTRY: Cat#T3983) at 25°C overnight, followed by incubation in CUBIC‐*R*+ for 1 day with gentle shaking. Images were acquired using either CQ1 or confocal microscopy (STELLARIS 8; Leica). 3D images were reconstructed by Imaris (OXFORD INSTRUMENTS). Other immunostaining was performed on frozen sections unless otherwise noted. Fresh tissue samples were fixed in 4% paraformaldehyde for 16 h and washed with PBS. Tissues were then placed in 15% sucrose in PBS for 16 h, followed by 30% sucrose in PBS for 24 h. They were embedded in Optimal Cutting Temperature (OCT) compound (SAKURA: Cat#45833). Frozen sections (5 μm thickness) were cut using a cryostat (Leica) and mounted on glass slides. The slides were washed three times with PBS. Sections were permeabilized with 0.3% Triton X‐100 (FUJIFILM: Cat#A16046) in PBS for 15 min at room temperature, followed by blocking with 5% normal goat serum for 1 h at room temperature to block non‐specific reactions. They were incubated with primary antibodies (Table S2) overnight at 4°C. After washing with PBS, they were incubated with appropriate fluorophore‐conjugated secondary antibodies (Table S2) for 1 h at room temperature. Nuclei were counterstained with 4′,6‐diamidino‐2‐phenylindole (DAPI) for 15 min at room temperature. Images were acquired using either a BZ‐X800 or STELLARIS 8.

### 2.5 Endometrial Epithelial Cell Height Analysis

The organoids on day 10 (before suspension culture) and day 13 (after suspension culture) were frozen, sectioned, and subjected to immunofluorescence staining. The maximum diameter of EpCAM‐positive cells in arbitrary sections was measured and defined as cell height as we reported previously [21]. Three independent experiments were performed using organoids derived from three patients, with three organoids examined per patient. Statistical significance was determined using a two‐sided Student's *t*‐test, with a *p* value of < 0.01.

### 2.6 Scanning Electron Microscopy

Organoids were fixed with 2% glutaraldehyde in a buffer (100 mm NaCl, 30 mm HEPES, and 2 mm CaCl _2_, pH 7.4) for 2 h at room temperature and postfixed with 1% osmium tetroxide in sodium cacodylate buffer (100 mM sodium cacodylate and 2 mm CaCl _2_, pH 7.0) for 2 h at 4°C. Osmicated samples were then dehydrated with a graded series of ethanol and infiltrated with t‐butanol (Wako, 028–03386; Sigma, 471712). All samples were freeze‐dried in a vacuum chamber at 0°C–5°C (VFD‐21S; Vacuum Device). Dried samples were coated with 5‐nm osmium in an osmium plasma coater (Neoc‐ST; Meiwa Fosis). Secondary electron images were acquired using a field‐emission scanning electron microscope (Quanta3D FEG, Thermo Fisher Scientific).

### 2.7 Hematoxylin and Eosin Staining

Hematoxylin and eosin (H&E) staining was performed on frozen sections to examine morphological structures as reported previously [26, 27, 28].

### 2.8 Statistics and Reproducibility

Statistical analysis was performed using R (version 4.5.0). All of the statistical methods are described in the figure legends. A two‐sided Student's *t*‐test was used for Figure 2B. Differences were considered significant at *p* values < 0.01.

## 3 Results

### 3.1 Development of a Novel Human Endometrial Organoid

**FIGURE 1:**
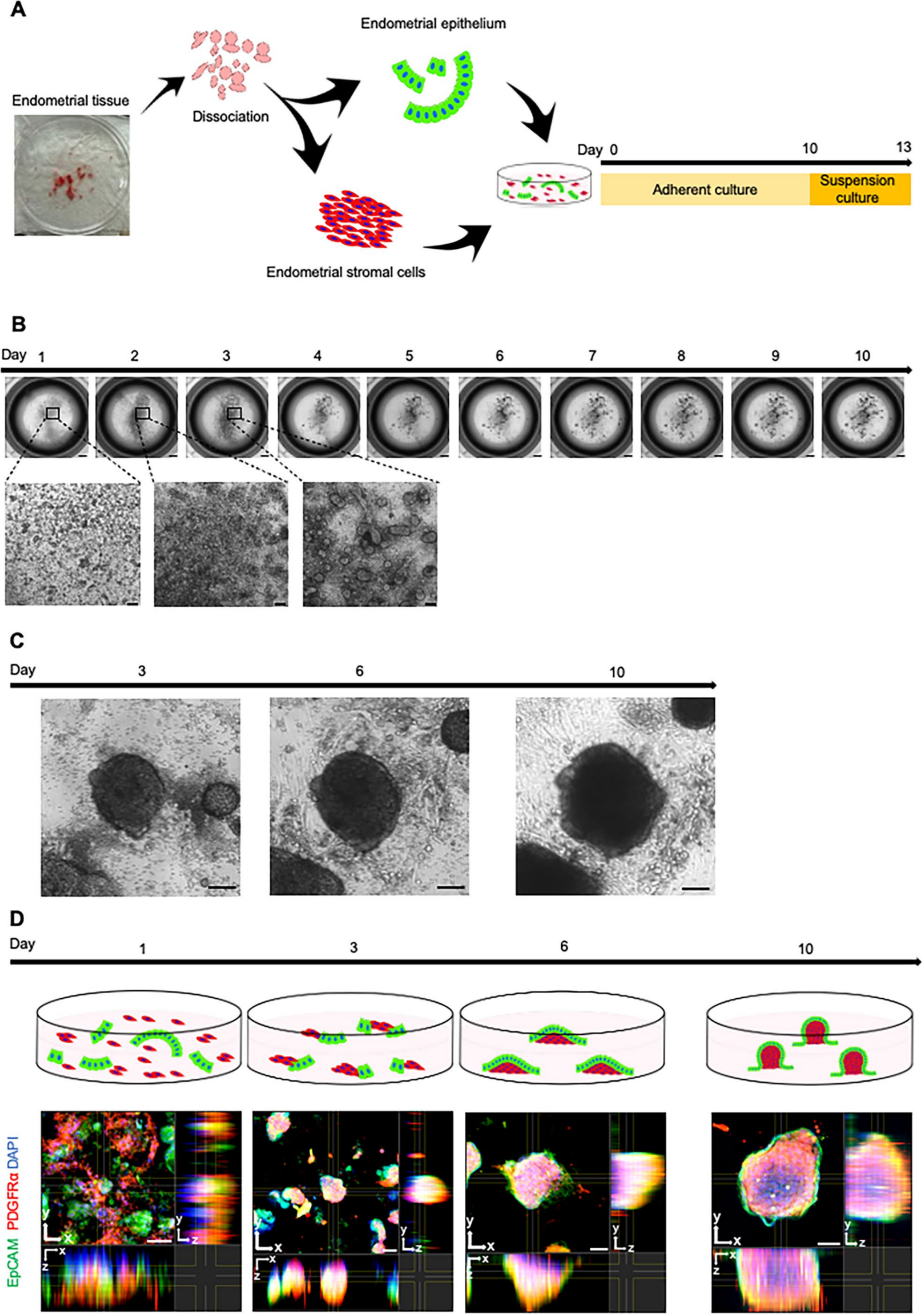
Self‐organized aggregate formation by adherent co‐culture of human endometrial epithelial and stromal cells. (A) Schematic illustration of the culture procedure used to generate human endometrial organoids. Endometrial epithelium and endometrial stromal cells (ESCs) were isolated from human endometrial tissues and co‐cultured under adherent conditions followed by suspension culture without extracellular matrix (ECM). (B) Representative bright field images of adherent co‐culture of endometrial epithelial cells (EECs) and ESCs. Spontaneous aggregation of EECs and ESCs was observed from day 3. Scale bars, 1000 μm. Higher‐magnification images of days 1, 2, and 3 are also shown. Scale bars, 100 μm. By day 10, the aggregates developed into three‐dimensional Ω‐shaped structures, with only their bottoms attached to the dish. (C) Higher‐magnification bright‐field images showing the morphological progression of aggregates during adherent culture. Scale bars, 50 μm. (D) Schematic and 3D immunostaining images of aggregates under adherent co‐culture. Representative cross‐sectional slices of aggregates in *x*–*y*, *x*–*z*, and *y*–*z* planes are shown. Images were obtained using confocal microscopy. EpCAM for EECs; PDGFRα for ESCs; DAPI for nuclei. Scale bars, 50 μm. They demonstrate the progressive spatial organization of EECs at the periphery and ESCs in the inner region. The localization of EECs and ESCs is shown in the schematic; EECs are shown in green and ESCs are shown in red.

Human EECs and ESCs were simultaneously seeded onto adherent plates using a medium previously established for mouse endometrial organoid culture [21] (Figure 1A). Endometrial tissues were enzymatically separated into epithelium‐rich and stromal fractions, and both fractions were combined in a proportional manner according to the number of epithelial glands used (see Methods). When tissues equivalent to > 6000 glands were seeded into a 24‐well plate, spontaneous aggregation of EECs and ESCs was observed from day 1, yielding small aggregates by day 3 (Figure 1B,C). In contrast, insufficient aggregation occurred with smaller tissue amounts (3000 glands) (Figure S1), indicating that a minimum input amount was required for aggregate formation. Immunostaining of day 3 aggregates revealed a disorganized distribution of PDGFRα‐positive ESCs and EpCAM‐positive EECs (Figure 1D, day 3). By day 6, the aggregates enlarged and the two cell populations became spatially organized, with ESCs occupying the inner region and EECs localizing to the periphery (Figure 1C,D, day 6). By day 10, they developed into three‐dimensional Ω‐shaped structures, attached to the culture dish only via their basal surface, with EECs forming an outer layer and ESCs filling the core (Figure 1C,D, day 10). This distinct epithelial–stromal architecture indicates that the aggregates were formed through self‐organization, a hallmark of organoid culture [10]. An adherent culture using conventional cell culture medium (DMEM with 10% FBS) instead of the organoid medium did not form aggregates (Figure S2), indicating that the organoid medium was essential for their formation. Furthermore, the adherent culture of EECs alone using organoid medium, without ESCs, did not lead to aggregation (Figure S3), indicating a critical role for ESCs in aggregate formation.

**FIGURE 2:**
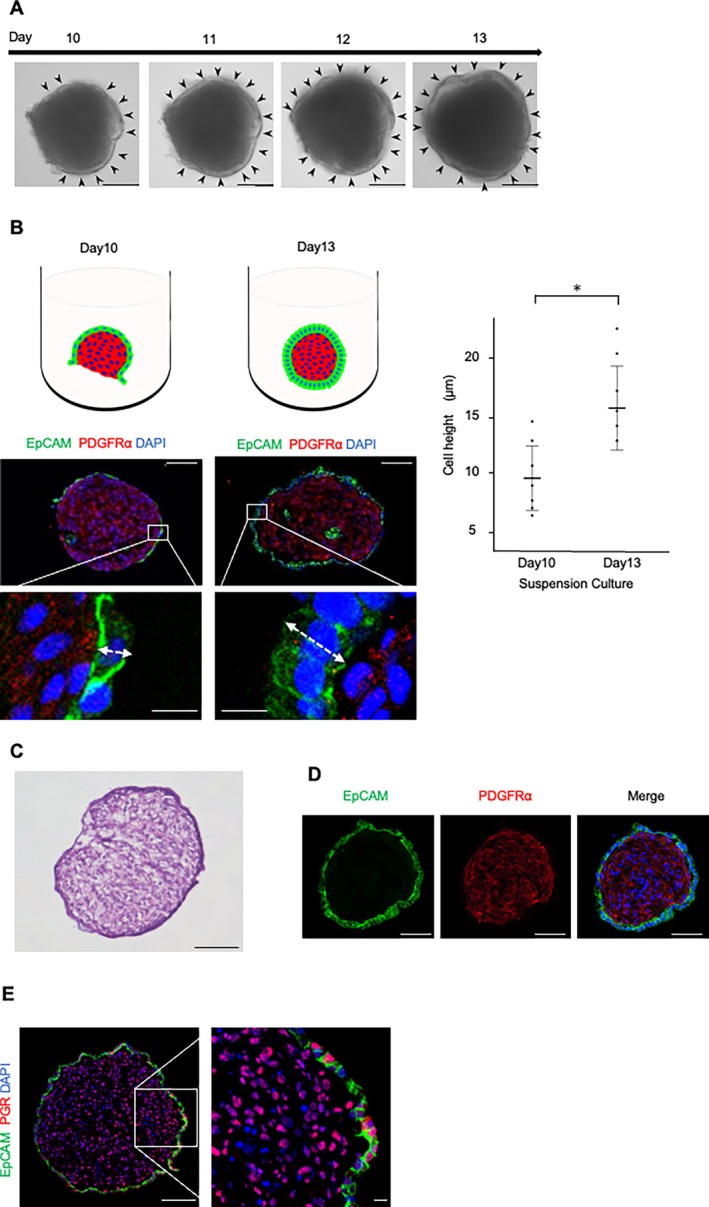
Suspension culture facilitates the formation of human endometrial organoids composed of EECs outside and ESCs inside. (A) Bright‐field images of suspension cultures of aggregates. The picked‐up aggregates were transferred to low‐attachment U‐bottom dishes for suspension culture. During the 3 days of suspension culture, a transparent epithelial layer covering the outer surface (indicated by dotted circles) gradually thickened and eventually enveloped the entire aggregate. Scale bars, 100 μm. (B) Structural changes of EECs induced by suspension culture. Top left, Schematic representation of epithelial coverage before (day 10) and after (day 13) suspension culture. Bottom left, Representative immunostaining images of the aggregates of frozen section of the aggregate are shown: EpCAM for EECs; PDGFRα for ESCs; DAPI for nuclei. Scale bars, 100 μm (low magnification), 20 μm (high magnification). Arrows indicate the measured height of EECs. Right, Quantification of the cell height of EECs before (day 10) and after (day 13) suspension culture. Data are presented as the mean ± SD of nine organoids from three patients (three organoids per patient). Data points are shown as dots. *p < 0.01 (two‐sided Student's *t* test). (C) Representative HE‐stained image for frozen section of the aggregate after suspension culture, showing a single epithelial layer surrounding a dense stromal core. Scale bars: 100 μm. (D) Representative immunostaining images for frozen section of the aggregate after suspension culture. E‐cadherin for EECs; Vimentin for ESCs; DAPI for nuclei. Scale bars, 100 μm. (E) Representative immunostaining images of a progesterone receptor (PGR) in frozen sections of the aggregate after suspension culture, demonstrating PGR expression in the majority of EECs and ESCs. EpCAM for EECs, DAPI for nuclei. Scale bars, 100 μm (low magnification), 10 μm (high magnification).

We next examined whether such aggregates could be generated from endometrial tissues obtained at different phases of the menstrual cycle. In most phases, small aggregates readily formed after seeding onto the plate as described above. However, when tissues obtained during the early proliferative phase were used, a single large aggregate formed as early as day 1 (Figure S4). Following a previously described mouse organoid culture protocol [21], the large aggregate was gently dissociated by pipetting it into smaller fragments and subsequently reseeded into the same culture dish, which resulted in the formation of small aggregates comparable to those from other phases. Thus, aggregates could be successfully generated from endometrial tissues irrespective of menstrual cycle phase. On day 10, the aggregates were picked up and transferred to low‐attachment U‐bottom dishes for suspension culture. During the first 3 days of suspension culture, a transparent layer covering the outer surface gradually thickened and eventually enveloped the entire aggregate (Figure 2A, arrowheads). The transparent layer was composed of EpCAM‐positive EECs (Figure 2B). During this process, squamous EECs became columnar (Figure 2B, top left) with a significant increase of cell height (Figure 2B, right). These results indicate that the suspension culture was essential for the generation of mature organoids covered with EECs.

Histological sections of the aggregates revealed a single outer layer of EpCAM‐positive EECs and an inner layer densely filled with PDGFRα‐positive ESCs (Figure 2C,D). Both EECs and ESCs were positive for the progesterone receptor (Figure 2E). On average, 6.4% ± 5.2% of epithelial cells (three cases; nine organoids total, mean ± SD) expressed FOXA2, a marker of glandular epithelial cells [29] (Figure S5), suggesting that most EECs of the organoid were composed of luminal epithelial cells. Taken together, these results show that we newly developed three‐dimensional human endometrial organoids with ESCs inside and EECs outside. Furthermore, the organoids were self‐organized and did not need to be embedded in an exogenous ECM.

### 3.2 The Organoids Exhibit Apical‐Out Polarity

**FIGURE 3:**
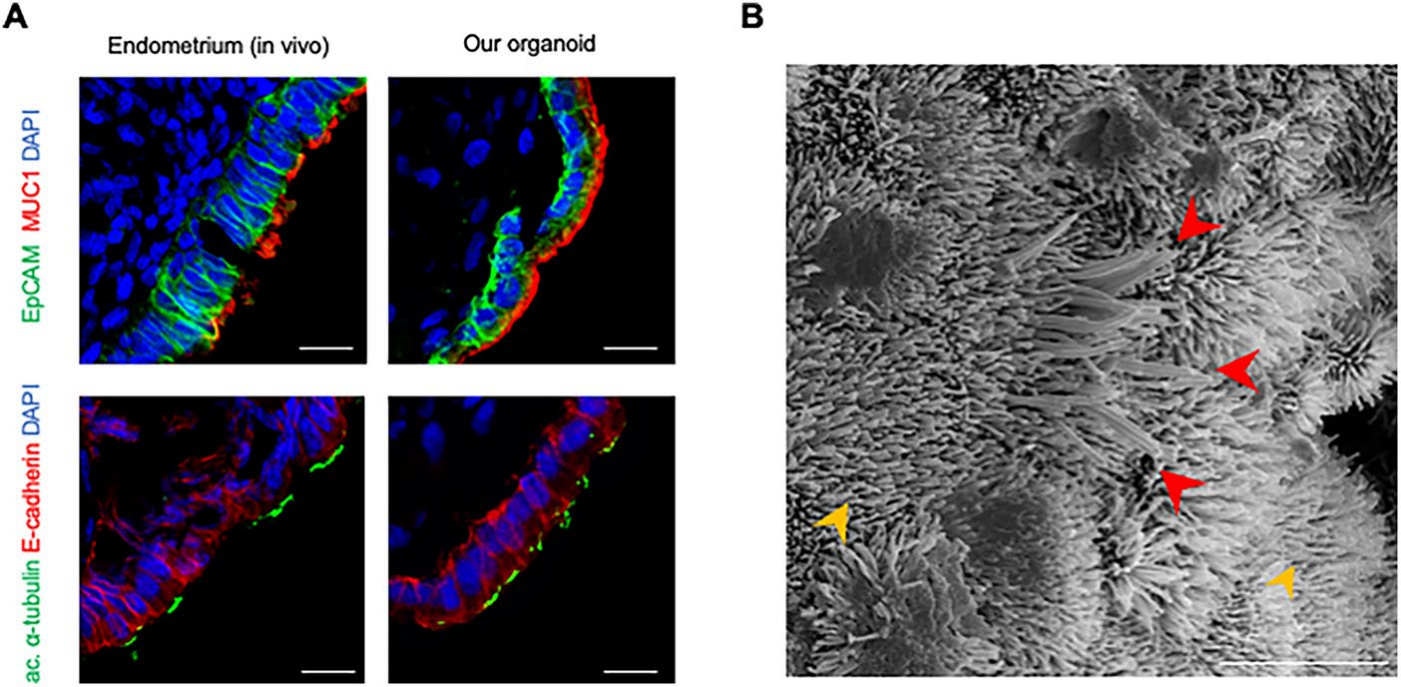
Novel human endometrial organoid exhibits apical‐out polarity. (A) Representative immunostaining images of MUC1 and acetylated α‐tubulin, proteins expressed on the apical side of luminal epithelial cells, in the human endometrium (left) and our organoid (right). In organoids, these apical markers are localized on the external surface of the epithelial layer, indicating apical‐out polarity. EpCAM and E‐cadherin for EECs; DAPI for nuclei. Scale bars, 20 μm. (B) Scanning electron microscopy revealed the presence of microvilli (yellow arrowheads) and cilia (red arrowheads), which are characteristic of the apical surface on the surface of the organoids. Scale bars, 5 μm.

Endometrial organoids, when co‐cultured with blastocysts, require apical‐out epithelial polarity to recapitulate the implantation process [30]. Therefore, we examined the polarity of our organoids. Mucin‐1 (MUC1) and acetylated α‐tubulin, which are enriched on the apical surface of luminal epithelial cells in vivo (Figure 3A, left), were strongly expressed on the external surface of EECs in our organoids (Figure 3A, right). In addition, scanning electron microscopy (SEM) observations demonstrated the presence of microvilli (yellow arrowheads) and cilia (red arrowheads) on the organoid surface (Figure 3B), both of which are characteristic features of the apical membrane. These findings indicate that the epithelial layer of our organoids exhibits apical‐out polarity, providing a useful model for investigating epithelial aspects of implantation in a blastocyst co‐culture setting. The organoids could be generated from endometrial tissues obtained at any menstrual cycle phase, including the menstrual phase (Table S1).

### 3.3 The Organoids Exhibit Hormonal Responsiveness

**FIGURE 4:**
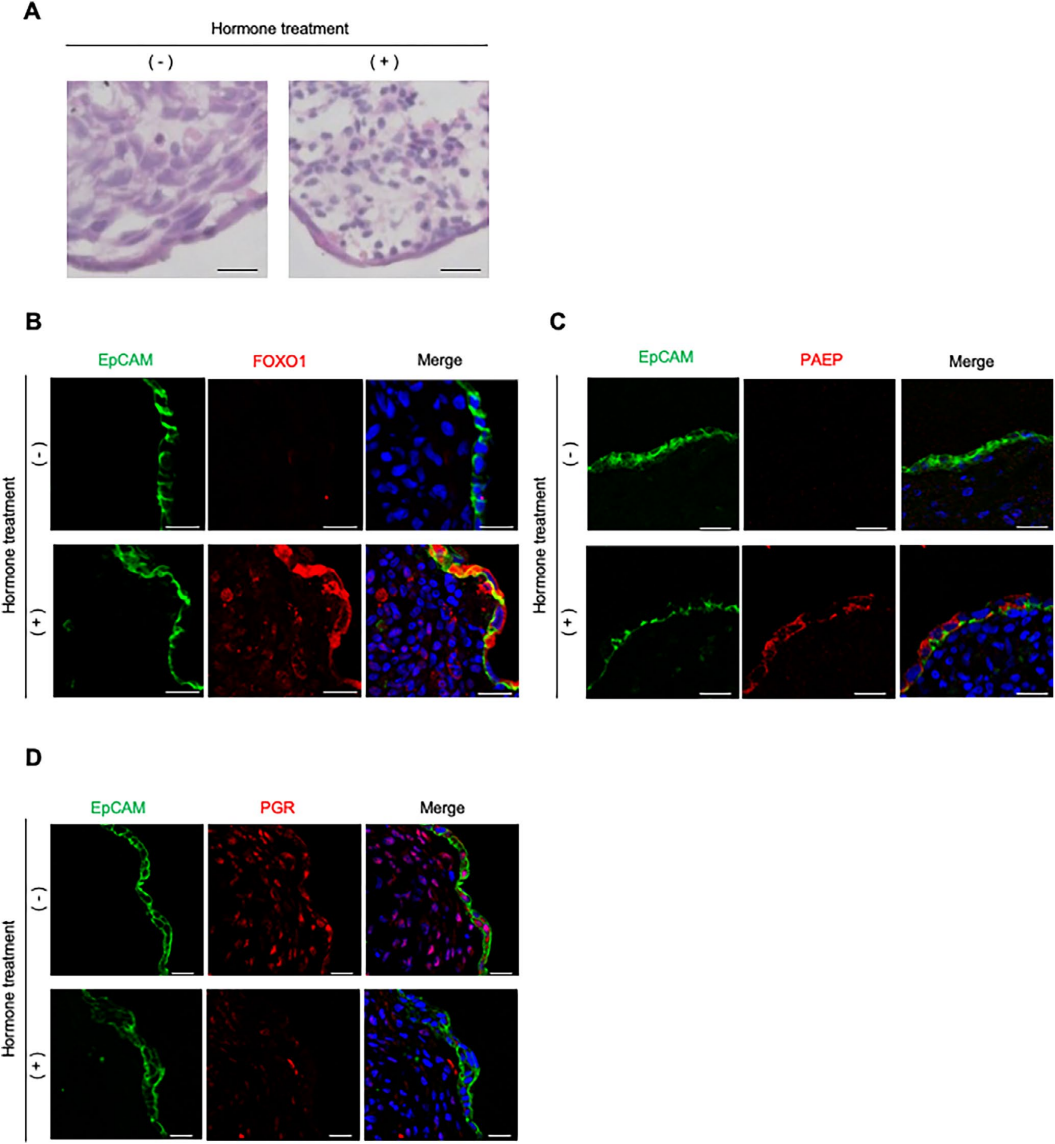
Novel human endometrial organoids exhibit hormonal responsiveness. (A) Representative HE‐stained images for frozen section of the organoid treated with (MPA + cAMP) or without hormonal treatment, showing decidualization‐like morphological changes in the stromal compartment upon hormonal treatment. Scale bars: 20 μm. (B) Representative images of FOXO1 for frozen section of the organoid treated with (MPA + cAMP) or without hormonal treatment, showing increased expression in both EECs and ESCs after hormonal stimulation. Scale bars, 20 μm. (C) Representative images of PAEP for frozen section of the organoid treated with (MPA + cAMP) or without hormonal treatment, showing increased expression in EECs after treatment. Scale bars, 20 μm. (D) Representative images of PGR for frozen section of the organoid treated with (MPA + cAMP) or without hormonal treatment, showing decreased expression in both EECs and ESCs after hormonal stimulation. Scale bars, 20 μm.

The human endometrium undergoes characteristic changes by progesterone during the implantation window to accept the blastocyst [31]. In particular, stromal cells undergo decidualization, exhibiting a cobblestone‐like morphology [32, 33]. When the organoids were treated with progesterone and cAMP, which are commonly used decidualization stimuli in vitro, similar morphological changes occurred in the stromal compartment (Figure 4A). Forkhead box O1 (FOXO1), a protein upregulated in both epithelial and stromal cells during the implantation phase [34], was markedly increased in EECs and ESCs upon stimulation (Figure 4B). Progestagen‐associated endometrial protein (PAEP), a marker elevated in luminal epithelial cells during the implantation window [35], was also upregulated in EECs (Figure 4C). In contrast, progesterone receptor (PGR), which is downregulated during this period [34, 36], decreased in both EECs and ESCs (Figure 4D). These findings demonstrate that our organoids recapitulate key implantation window‐specific hormonal responses in both EECs and ESCs.

## 4 Discussion

The detailed mechanisms of human implantation have not been fully elucidated because the process occurs within the concealed uterine environment. In addition, obtaining human endometrial tissue from the implantation site is difficult due to ethical problems. Therefore, the development of an endometrium‐like structure that closely mimics the in vivo environment and its application to in vitro implantation studies have long been anticipated. We previously established novel mouse endometrial organoids and successfully reproduced in vitro implantation by co‐culturing them with mouse blastocysts [21]. In the present study, by adapting a similar approach for human tissues, we successfully developed a novel human endometrial organoid with four notable features: (1) the epithelium exhibits apical‐out polarity; (2) the inner region contains abundant ESCs; (3) the organoids can be formed without exogenous ECM embedding; and (4) both EECs and ESCs display hormone responsiveness. To our knowledge, this is the first human endometrial organoid that simultaneously exhibits all these characteristics.

Conventional human endometrial epithelial organoids are generated under 3D conditions by embedding EECs into ECM such as Matrigel [11, 22]. In contrast, our organoids were formed through the spontaneous three‐dimensional aggregation of EECs and ESCs under adherent culture conditions, without the use of exogenous ECM. This phenomenon was not observed when EECs were cultured alone in organoid medium, indicating the essential role of ESCs in the aggregation process. Spontaneous aggregation between tissue‐specific epithelial cells and mesenchymal stem cells has been reported in adherent co‐culture systems [37, 38]. Because human ESCs possess mesenchymal stem cell‐like properties [39], it is likely that our organoids were formed through a similar aggregation mechanism. Furthermore, the resulting aggregates exhibited a spatial organization, with EECs localized to the outer side and ESCs to the inner inside. The initial adherent culture step likely facilitated the cell type‐specific localization based on the intrinsic properties of each cell type. Such establishment of cell type‐specific localization is a distinctive feature of organoid systems, referred to as self‐organization [10]. Notably, when EECs and ESCs were co‐cultured in conventional culture medium (DMEM containing 10% FBS) instead of organoid medium, aggregates failed to form, supporting the idea that self‐organization is the driving force behind our organoid formation. Self‐organized aggregates exhibit tissue structures and functions that more closely resemble those observed in vivo [40]. Therefore, in our organoid, not only were EECs and ESCs arranged in a physiologically relevant manner, but the EECs also exhibited apical‐out polarity, with their apical surface oriented away from the ESCs, as seen in vivo. Previous studies have shown that conventional apical‐in endometrial organoids embedded in ECM can reverse their polarity to apical‐out when they were cultured under ECM‐free conditions [20]. Thus, the absence of an exogenous ECM in our culture system may also have contributed to the spontaneous acquisition of apical‐out polarity.

To use the organoids as an in vitro implantation model, they must be completely covered by EECs. It was reported that aggregates composed solely of ESCs accepted blastocyst attachment in 100% of the cases [19], which does not reflect the limited implantation efficiency observed in humans [41, 42]. These findings highlight the importance of the epithelial layer for proper blastocyst attachment. In our model, immediately after detachment from the monolayer culture (Figure 2A, day 10), the organoid was not fully covered by EECs. However, after 3 days of suspension culture, EECs completely covered the exposed stromal surface and transformed into columnar epithelial cells resembling those in vivo. Therefore, the suspension culture step was crucial for generating organoids suitable for implantation studies, providing complete epithelial coverage.

Unlike conventional human endometrial organoids, our organoids contain ESCs inside. Because epithelial‐stromal interactions play an important role during implantation [43, 44], our model may be able to reproduce such interactions in vitro. In addition, during the implantation process, ESCs undergo decidualization and exhibit numerous functional changes [12, 13, 14, 16, 17, 45]. Decidualization regulates trophoblast invasion [15] and contributes to pregnancy maintenance [46, 47]. Therefore, organoids incorporating ESCs are essential for recapitulating the in vivo implantation process. Furthermore, our organoids were formed by cell self‐aggregation without the use of exogenous ECM, which facilitated close cell‐to‐cell contact and the formation of a dense stromal layer beneath the epithelial layer. This architecture will enable observation of the interactions between blastocysts and ESCs during the in vitro implantation process. Although two previous studies have reported human endometrial organoids containing both EECs and ESCs embedded in ECM [18, 19], those organoids did not reproduce a dense ESC layer, probably due to reduced cell density caused by the presence of ECM.

Another important characteristic needed for reproducing endometrial function is responsiveness to hormonal stimulation. The human endometrium in vivo is precisely regulated by estrogen and progesterone, which induce structural and functional changes to accept the blastocyst [31, 48]. In our organoids, hormone treatment successfully recapitulated the expression pattern of implantation‐related markers in both EECs and ESCs (FOXO1, PAEP, and PGR), as well as the decidualization‐like morphological changes in ESCs. In contrast, stromal responsiveness to hormones was not observed in the organoids reported by Shibata et al. [19]. Therefore, a notable advantage of our organoids is their ability to reproduce hormone responsiveness in both EECs and ESCs.

Our organoids could be generated from endometrial tissues obtained at any menstrual cycle phase, including the menstrual phase. In contrast, conventional epithelial organoids were not generated from menstrual‐phase tissues [11, 19, 22]. This is a highly advantageous feature for protocols utilizing precious human specimens. One point to note is that when endometrial tissues from the early proliferative phase were used, dynamic large aggregation formed. By gently disassembling these large aggregates into smaller fragments, as we previously did for mouse organoid formation [21], organoids were successfully developed even from early proliferative‐phase tissues.

A limitation of this study is that organoid generation requires a relatively large amount of endometrial tissue, enough to contain approximately 6000 glands. Considering that the amount of endometrial tissue obtainable by biopsy from women with infertility is limited, further improvements are needed to enable organoid generation from smaller tissue samples. Another limitation is that, among the various cell types constituting the in vivo endometrium, our organoids lack glandular epithelial cells, vascular endothelial cells, and immune cells [49]. Establishing culture conditions that allow their maintenance within organoids will be an important future challenge. In addition, during the implantation window in vivo, FOXO1 is expressed in both the cytoplasm and nucleus of EECs and ESCs, with stronger nuclear localization [34]. However, in our organoids, nuclear FOXO1 staining was relatively weak, which may reflect incomplete induction of an implantation‐phase endometrium in vitro and suggests that additional hormonal treatments may be required for full implantation‐phase induction. Finally, it will be necessary to verify whether our organoids can recapitulate implantation in vitro by co‐culturing them with surplus human blastocysts or blastoids derived from induced pluripotent stem cells [50, 51, 52]. Unlike the assembloids reported by Shibata et al. [19], our organoids do not contain glandular epithelial cells. Considering that, in addition to luminal epithelial cells, glandular epithelium also plays important roles in implantation [29, 53], our organoids are not intended to fully recapitulate the in vivo implantation process. Further improvements to integrate glandular epithelial cells into the organoids will be required. These experiments will be important next steps for future research.

In conclusion, we established a human endometrial organoid that reproduces the in vivo architecture and hormonal responsiveness of the endometrium. The organoid was formed through self‐organization of EECs and ESCs under ECM‐free adherent culture conditions and can be generated from tissues obtained at any menstrual phase. This model provides a physiologically relevant platform for studying human implantation and may contribute to elucidating the mechanisms underlying implantation failure.

## Funding

This work was supported by Japan Society for the Promotion of Science, 23K27734, 24K12533, 24K12579, 23K15838, 25K24017, JST FOREST, JPMJFR245C.

## Disclosure

Human rights statements: All procedures followed were in accordance with the ethical standards of the responsible committee on human experimentation (institutional and national) and with the Helsinki Declaration of 1964 and its later amendments.

## Ethics Statement

This study was conducted in accordance with the Declaration of Helsinki and approved by the Institutional Review Board of Yamaguchi University Hospital.

## Consent

Informed consent was obtained from all the patients in this study.

## Conflicts of Interest

The authors declare no conflicts of interest.

## Supporting information


**Appendix S1:** rmb270030‐sup‐0001‐AppendixS1.docx.
**Figure S1:** rmb270030‐sup‐0001‐AppendixS1.docx.
**Figure S2:** rmb270030‐sup‐0001‐AppendixS1.docx.
**Figure S3:** rmb270030‐sup‐0001‐AppendixS1.docx.
**Figure S4:** rmb270030‐sup‐0001‐AppendixS1.docx.
**Figure S5:** rmb270030‐sup‐0001‐AppendixS1.docx.
**Table S1:** Clinical characteristics of patients used for organoid derivation.
**Table S2:** Antibody details for immunofluorescence labeling.

## Data Availability

All data generated or analyzed during this study are included in this published article and its supporting information files.
