# Supplementary material for: Establishment of a Novel Human Endometrial Organoid
Source: Reprod Med Biol. 2026 Feb 16;25(1):e70030. doi: 10.1002/rmb2.70030 (PMC12909605; doi:10.1002/rmb2.70030)
Supplement: Supplementary file 1 — Appendix S1: rmb270030‐sup‐0001‐AppendixS1.docx. Figure S1: rmb270030‐sup‐0001‐AppendixS1.docx. Figure S2: rmb270030‐sup‐0001‐AppendixS1.docx. Figure S3: rmb270030‐sup‐0001‐AppendixS1.docx. Figure S4: rmb270030‐sup‐0001‐AppendixS1.docx. Figure S5: rmb270030‐sup‐0001‐AppendixS1.docx. Table S1: Clinical characteristics of patients used for organoid derivation. Table S2: Antibody details for immunofluorescence labeling. [file RMB2-25-e70030-s001.docx]

**Supplementary Information**

**
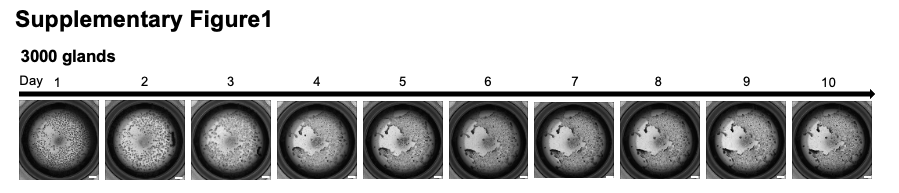
**

**Supplementary Figure 1.**

Representative bright field images of adherent co-culture of EECs and ESCs derived from endometrial tissues equivalent to 3,000 glands. Spontaneous aggregation was not observed. Scale bars, 1000 µm.

**
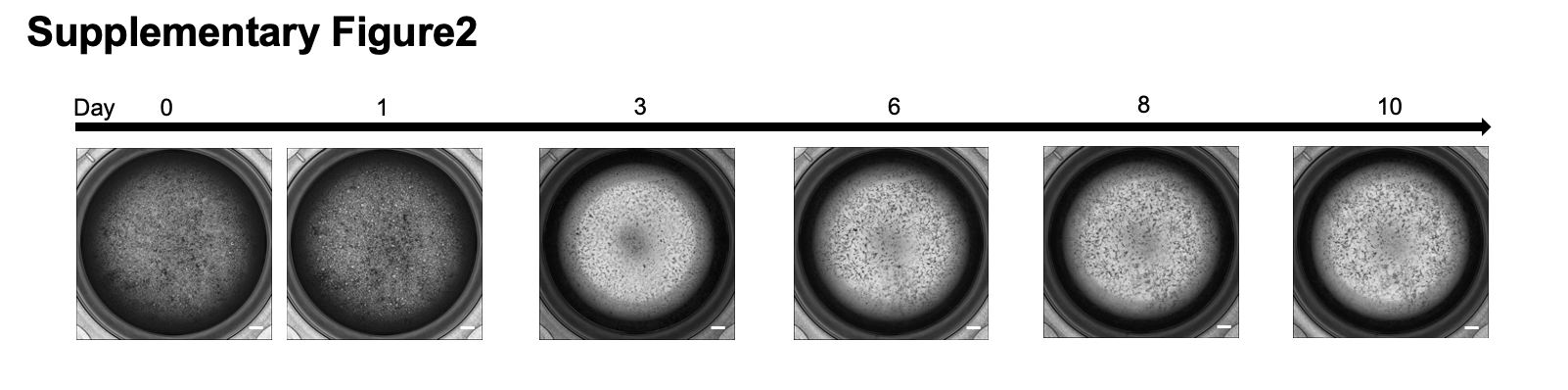
**

**Supplementary Figure 2.**

Representative bright field images of adherent co-culture of EECs and ESCs in conventional culture medium (DMEM with 10% FBS). Spontaneous aggregations of EECs and ESCs were not observed. Scale bars, 1000 µm.

**
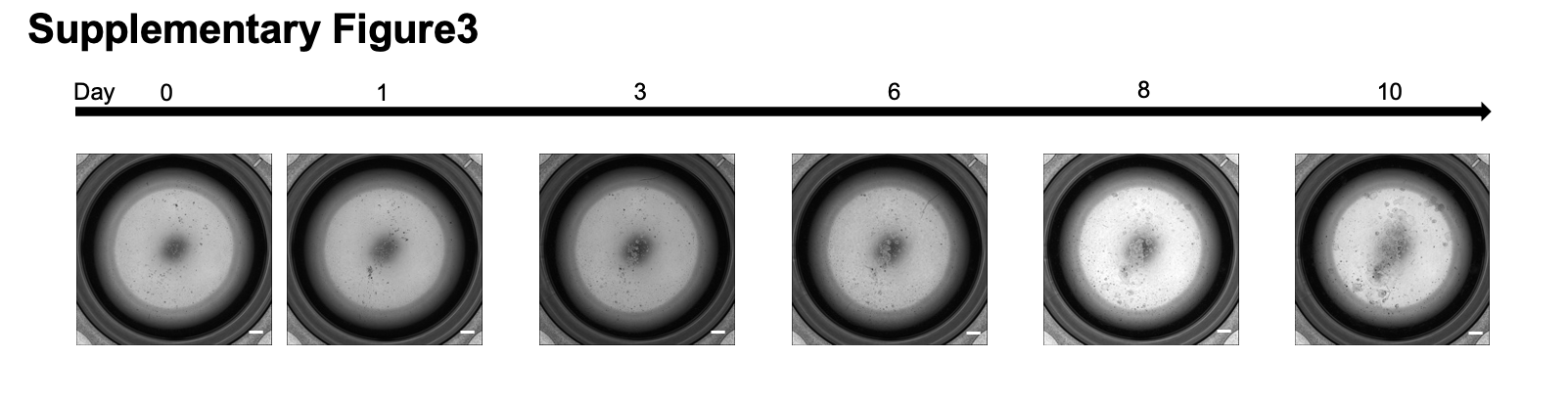
**

**Supplementary Figure 3.**

Representative bright field images of adherent culture of EECs without ESCs. Spontaneous aggregations were not observed. Scale bars, 1000 µm.

**
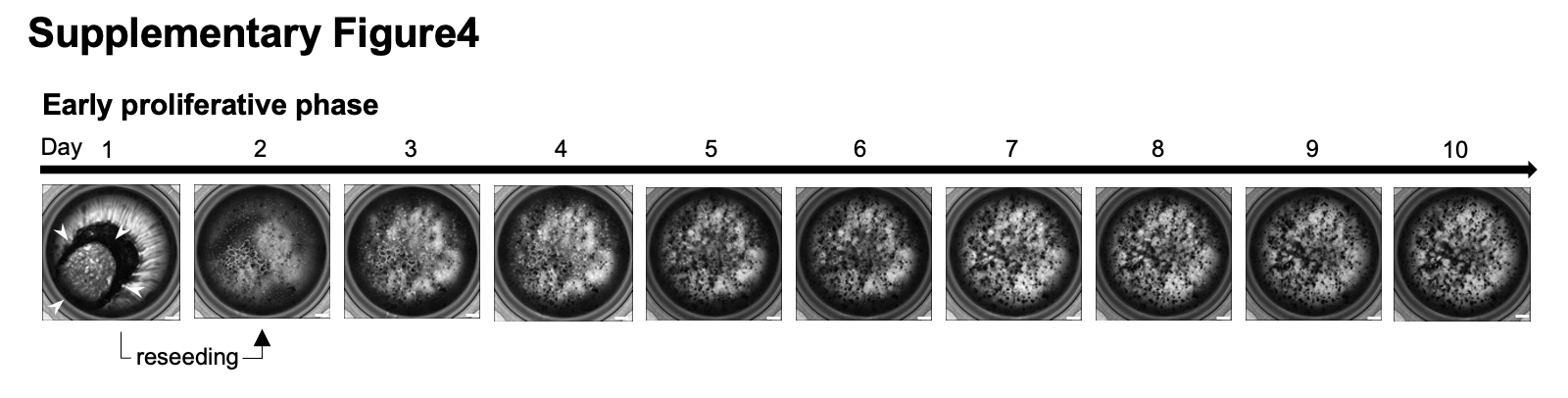
**

**Supplementary Figure 4.**

Representative bright field images of adherent co-culture of EECs and ESCs obtained during the early proliferative phase. A single large aggregate formed on day 1 (arrowheads). This aggregate was gently dissociated by pipetting into smaller fragments and subsequently reseeded into the same culture dish. By day 10, small aggregates comparable to those derived from other phases had formed. Scale bars, 1000 µm.


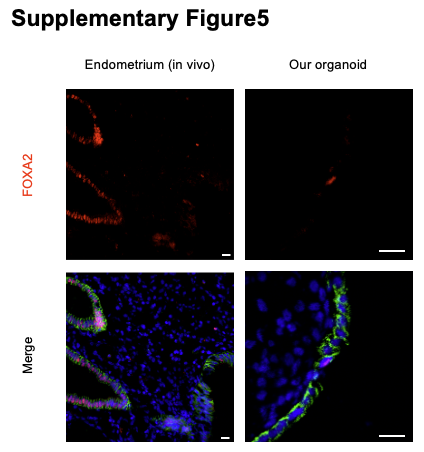


**Supplementary Figure 5.**

Representative immunostaining images of the glandular epithelial marker FOXA2 in our organoid (right). Human endometrium was used as a positive control (left). EpCAM for EECs; DAPI for nuclei. Scale bars: in vivo, 20µm; organoid, 20µm. The number of FOXA2-positive cells among EECs in a single section of the organoid was counted, and the percentage of FOXA2-positive cells was calculated. On average, 6.4 ± 5.2% (mean ± SD of three cases, each averaged from three organoids) of epithelial cells were FOXA2-positive, indicating that the remaining EECs corresponded to luminal epithelial cells.

Supplementary Table 1. Clinical characteristics of patients used for organoid derivation

| **Case ID** | **Age(years)** | **Surgical indication** | **Menstrual cycle** |
| --- | --- | --- | --- |
| Case_1 | 51 | Uterine leiomyoma | Early proliferative phase |
| Case_2 | 40 | Uterine leiomyoma | Menstrual phase |
| Case_3 | 50 | Uterine leiomyoma | Early proliferative phase |
| Case_4 | 46 | Uterine leiomyoma | Mid-secretory phase |
| Case_5 | 37 | Uterine leiomyoma | Late proliferative phase |
| Case_6 | 41 | Uterine leiomyoma | Menstrual phase |
| Case_7 | 42 | Endometrial polyp | Early proliferative phase |
| Case_8 | 41 | Cervical cancer,  FIGO stage IA1 | Early secretory phase |
| Case_9 | 48 | Uterine leiomyoma | Early secretory phase |
| Case_10 | 47 | Endometrial polyp | Early secretory phase |
| Case_11 | 46 | Uterine leiomyoma | Menstrual phase |
| Case_12 | 44 | Uterine leiomyoma | Late proliferative phase |
| Case_13 | 47 | Uterine leiomyoma | Late proliferative phase |
| Case_14 | 42 | Uterine leiomyoma | Mid-secretory phase |
| Case_15 | 39 | Cervical cancer,  FIGO stage IA1 | Late proliferative phase |
| Case_16 | 48 | Uterine leiomyoma | Late proliferative phase |
| Case_17 | 35 | Endometrial polyp | Menstrual phase |
| Case_18 | 45 | Endometrial polyp | Late secretory phase |

Supplementary Table 2. Antibody details for Immunofluorescence labeling

| **Target** | **Host** | **Dilution** | **Supplier(Catalog.#)** |
| --- | --- | --- | --- |
| Primary Antibodies |  |  |  |
| EpCAM | Mouse | 1:800 | Cell Signaling Technology(2929) |
| PDGFRα | Rabbit | 1:500 | Cell Signaling Technology(3174) |
| PGR(Progesterone receptor) | Rabbit | 1:100 | Abcam(ab16661) |
| MUC1 | Rabbit | 1:250 | Abcam(ab109185) |
| Acetylated tubulin | Mouse | 1:500 | Sigma Aldrich(T7451) |
| E-cadherin | Rabbit | 1:200 | Cell Signaling Technology(3195) |
| FOXO1 | Rabbit | 1:100 | Cell Signaling Technology(2880) |
| PAEP | Rabbit | 1:500 | Atlas Antibodies(HPA029473) |
| FOXA2 | Rabbit | 1:400 | Cell Signaling Technology(8186) |
| Secondary Antibodies |  |  |  |
| Anti-Mouse, Alexa Fluor 488-conjugated | Goat | 1:1000 | Abcam(150113) |
| Anti-Rabbit, Alexa Fluor 594-conjugated | Goat | 1:1000 | Abcam(150084) |
